# A Spore-Based Biosensor-on-Pillar Platform for Detecting β-Lactam Antibiotics in Milk

**DOI:** 10.3390/molecules31091436

**Published:** 2026-04-26

**Authors:** Sammer UƖ Hassan, Zhuoxin Liu, Prashant Goel, Naresh Kumar, Xunli Zhang

**Affiliations:** 1 School of Engineering, University of Southampton, Southampton SO17 1BJ, UK; sammer@drhassantutoring.co.uk (S.U.H.); zl3666@columbia.edu (Z.L.); 2 Department of Biomedical Engineering, Columbia University, New York, NY 10027, USA; 3 Dairy Microbiology Division, ICAR-National Dairy Research Institute, Karnal 132001, Haryana, India; prashant318goyal@gmail.com

**Keywords:** miniaturization, antimicrobial resistance, antibiotics, β-lactam, colorimetric

## Abstract

Antimicrobial resistance (AMR) is increasingly becoming a major global public health concern, as antibiotics are losing their effectiveness at an alarming rate due to drug resistance. The β-lactam group of antibiotics are widely used in dairy farms to treat animal infections, and their presence in the food chain is a significant concern. Addressing this issue requires the development of effective analytical tools for the rapid detection of antibiotics. In this work, a miniaturized Biosensor-on-Pillar platform was developed for detecting β-lactam antibiotics in milk, which operates in a rapid, cost-effective, and user-friendly format, making it particularly suitable for resource-limited settings. The platform employs an enzyme induction-based approach, wherein *Bacillus cereus* spores germinate in the presence of β-lactam antibiotics, leading to the production of β-lactamase enzyme, which is then recognized using a chromogenic substrate functionalized on paper associated with the pillar platform. The developed biosensor can detect 12 β-lactam antibiotics with limits of detection (LODs) ranging from 1 to 1000 ppb, achieving sensitivity at or below the maximum residue limits (MRLs) set by regulatory bodies (FSSAI/CODEX) for the majority of the tested antibiotics. The performance of the platform, including the design, fabrication, and working principle, was further evaluated by analyzing six blind milk samples, yielding significant results compared to the commercially available AOAC-approved gold-standard method. Hence, the developed biosensor demonstrates promising potential for the rapid, cost-effective and high-throughput screening of milk samples for β-lactam antibiotics, benefiting the dairy industry and ensuring food safety.

## 1. Introduction

Antimicrobial resistance (AMR) represents a global public health concern, as antibiotics are increasingly becoming ineffective mainly due to drug resistance, making the treatment of infectious diseases more challenging [1,2,3]. This issue is primarily associated with the misuse of antibiotics in humans and animals, which accelerates resistance development. In dairy farming, the β-lactam group of antibiotics are widely used, particularly for treating mastitis, dry cow therapy, metritis, and infections in the respiratory tract, prostate, urinary tract, skin, and soft tissues [4,5]. Consequently, the presence of β-lactam antibiotics in the food chain is a significant concern, as they can hinder the growth of starter cultures used in the dairy industry, trigger allergic reactions in susceptible individuals, and disrupt intestinal flora [6]. Many of these antibiotics have been classified as “critically important” by the World Health Organization [7]. Recently, the Food Safety and Standards Authority of India (FSSAI) specified maximum residue limits (MRLs) for antibiotic residues in milk to ensure consumer safety. Despite these regulatory frameworks, monitoring compliance at the farm and processing level remains challenging. Conventional reference methods such as LC-MS and HPLC, while highly accurate, require complex sample preparation, intricate analytical procedures, and a skilled workforce for operation [8]. Therefore, developing cost-effective and easy-to-use testing is crucial for ensuring regulatory compliance and detecting these contaminants in dairy products, particularly in resource-limited settings.

Early methods for testing antimicrobial residues in milk were based on microbial inhibition, dating back to 1952 [6]. Since then, several microbial growth inhibition methods have been developed, varying in test organisms, indicators, incubation conditions, detection range, and sensitivity [6]. Alternative approaches for detecting β-lactam antibiotics involve receptor proteins [6,9]. For example, Boehle et al. developed a microfluidic paper-based analytical device (µPAD) using enzyme competition with nitrocefin as a chromogenic substrate [10]. More recently, *Bacillus cereus* spores have been employed as an indicator strain for the real-time detection of β-lactam antibiotics in milk [11,12], with the concept further extended to detect a range of antibiotics [4,13,14] and pesticides in milk [15,16].

Using spore germination inhibition, colorimetric detection has been achieved with Dipicolinic Acid (DPA) assays [13,17], later adapted into paper strip formats based on marker enzyme release. The development of a blue color on the paper strip visually indicates the absence of antibiotics after incubation for 60 min at 64 °C [18]. This technology has been validated by an NABL-accredited laboratory and demonstrates strong performance in sensitivity, selectivity, shelf stability, detection time, cost, and portability. However, the reliance on visual color interpretation limits its ability to provide quantitative results.

Recent advances in the development of Lab-on-a-Chip miniaturized devices have led to the integration of microanalysis platforms [19,20]. The fabrication of these miniaturized devices involves materials such as glass, silicon, polystyrene, and paper. These advances have enabled the development of portable, low-cost analytical tools for food safety monitoring.

**Figure 1:**
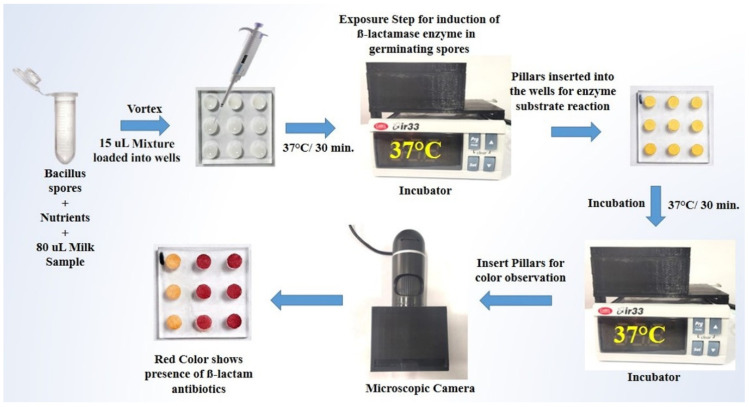
Workflow of the Biosensor-on-Pillar platform, including pillar/well chips, a homemade incubator, and a miniaturized microscopic imaging system.

The present study aimed to develop a Biosensor-on-Pillar system for detecting β-lactam antibiotics in milk, building on our extensive work on spore enzyme induction by antibiotics with a colorimetric readout [11,21,22] (Figure 1). *Bacillus cereus* was employed for the production of β-lactamase enzyme, induced by β-lactam antibiotics, with nitrocefin used as a chromogenic indicator for their presence. A plastic microchip with a nine-pillar/well configuration was designed to facilitate paper–milk interaction, enabling the simultaneous analysis of nine samples in a single run for high-throughput testing. This multiplexed format, combined with a custom-built miniaturized incubator and a digital imaging system, represents a significant advance over our previous single-use paper-strip system [11], providing integrated, quantitative, and portable capabilities suitable for dairy industry and field deployment. Additionally, a miniaturized incubator and a digital imaging system were custom-built using 3D printing technology to accommodate the microchip for incubation and record the colorimetric readout. Following a demonstration of the system’s robustness using pure β-lactamase enzyme and nitrocefin, assay conditions were optimized for detecting β-lactam antibiotics in milk. The system’s performance was then validated against a gold-standard method. Finally, a protocol was established for determining the limit of detection (LOD) for β-lactam antibiotics in milk.

## 2. Results and Discussion

### 2.1. Characterization of Biosensor-on-Pillar Chips

**Figure 2:**
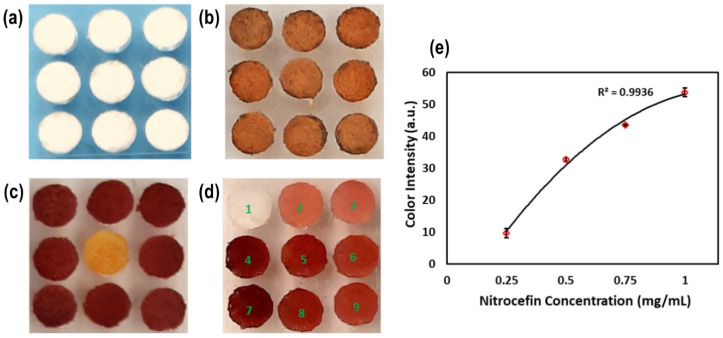
(**a**) Filter paper attached to the pillars. (**b**) The chromogenic substrate nitrocefin dispensed on paper. (**c**) Nitrocefin-loaded pillars dipped into the β-lactamase sample, except for the middle yellow one dipped into the water only. (**d**) Different concentrations of nitrocefin (pillar **1**—0 mg/mL; **2** and **3**—0.25 mg/mL; **5** and **6**—0.5 mg/mL; **8** and **9**—0.75 mg/mL; **4** and **7**—1.0 mg/mL) loaded on paper and dipped into β-lactamase samples. (**e**) Variation of color intensity with different concentrations of nitrocefin measured via the miniaturized imaging system.

Pure β-lactamase enzyme and the chromogenic substrate nitrocefin were used to quantify the color development on the test paper on a pillar with a nitrocefin concentration ranging from 0.25 mg/mL to 1.0 mg/mL. Figure 2a shows the dispensing of the reagents on the paper attached to the pillars. The variation in color intensity after reagent loading between each paper was found to be 2% (% RSD), indicating high uniformity in sensor preparation. Furthermore, the chromogenic substrate nitrocefin was dispensed on paper and dipped into the pure β-lactamase enzyme samples to generate a red color (Figure 2c,d). Variations of color intensity with different concentrations of nitrocefin were measured with miniaturized imaging (Figure S2), and the results are plotted in Figure 2e (n = 3). Notably, the correlation between nitrocefin concentration and color intensity (Figure 2e) yielded an R^2^ value of 0.9936, demonstrating strong consistency in the sensor response. The results clearly demonstrated the correlation of color change of the test paper with different concentrations of chromogenic reagent.

### 2.2. Optimization of Assay Conditions

**Figure 3:**
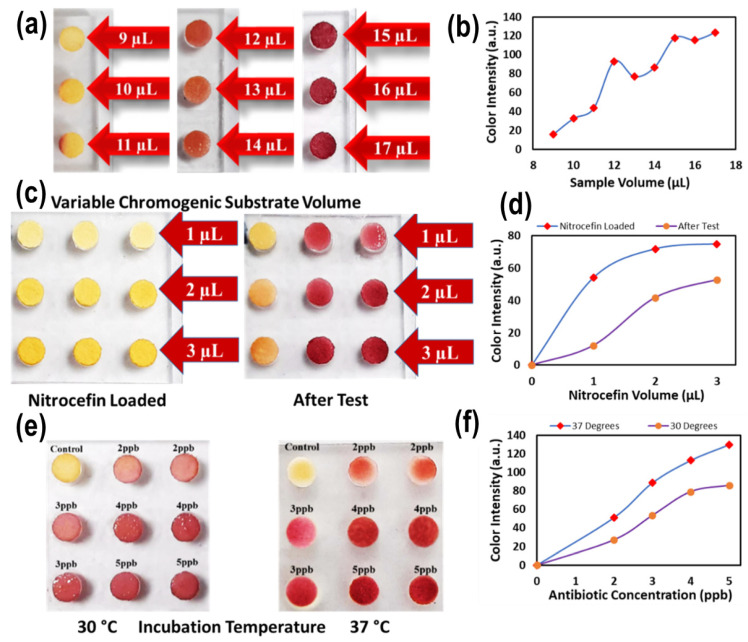
(**a**) Optimization of the sample volume (7–17 µL). Red color development on the paper shows the enzyme–substrate reaction, while a yellow color shows no reaction. (**b**) Variation of color intensity with different sample volumes. (**c**) Optimization of the amount of the chromogenic substrate nitrocefin used for functionalizing the test paper on pillars by varying the substrate solution volume (1–3 µL) (pillars in the left column are controls and the values plotted are an average of two runs). (**d**) Variation of color intensity with different nitrocefin volumes. (**e**) Optimization of incubation temperature (30 °C and 37 °C). (**f**) Variation of color intensity at different temperatures (the values plotted are an average of two runs).

A range of key operating parameters were optimized with the Biosensor-on-Pillar platform for the detection of β-lactam antibiotics in milk, including the sample volume containing antibiotic and bacillus spores, chromogenic substrate volume, and incubation temperature. Figure 3a,b depict the color changes on pillar-supported test papers where sample volumes in the wells varying from 7 to 17 µL were loaded into wells of the well chip. This shows that, overall, the red color development on the paper indicated the detection of antibiotics in milk as a result of the enzyme/substrate reaction. In contrast, the yellow color suggested either no reaction or an undetectable reaction. By varying the sample volume, this shows that a sample volume of 15 µL was sufficient to yield full-color development on the test paper (on pillars) while avoiding the well becoming dry during incubation. Adding more sample volume (>15 µL) resulted in overflow when pillars were introduced into the wells for incubation. Therefore, a 15 µL sample volume was selected for the subsequent tests in this study.

The amount of the chromogenic substrate nitrocefin used for functionalizing the test paper was optimized by varying the solution volume (1–3 µL) on the paper attached to the pillars (Figure 3c). The results revealed an upward trend in color intensity with increased substrate volume (Figure 3d). The color development with a substrate volume of 3 µL produced the highest intensity, where a red color still developed but in lighter intensity at lower concentrations. Therefore, a 3 µL substrate volume was finalized for further experiments to functionalize the test paper anchored on the pillars.

It is important to control and optimize the incubation temperature with the miniaturized incubator developed, as the spore germination and enzymatic–substrate reaction is temperature-dependent [23]. The results are shown in Figure 3e,f at two temperature levels of 30 °C and 37 °C. The selection of 37 °C as the optimal incubation temperature is primarily based on it being the optimal growth temperature of *B. cereus*, which promotes efficient spore germination and β-lactamase induction [23]. The higher color intensity observed at 37 °C compared to 30 °C is a consequence of enhanced enzymatic activity at the organism’s optimal temperature, not the primary selection criterion. A further increase in temperature above 37 °C was not explored, as higher temperatures risk denaturing the enzyme and impairing spore viability. Therefore, the incubation temperature of 37 °C was chosen for assay development and further investigations.

### 2.3. Optimized Assay Protocol

Based on the optimization of the key operating parameters, an assay protocol was developed with the Biosensor-on-Pillar platform for detecting β-lactam antibiotics in milk. The protocol consisted of two main steps: (i) exposure of Bacillus spores to β-lactam antibiotics for the induction of β-lactamase enzyme, and (ii) the enzyme–substrate reaction.

Step 1—Exposure: A total of 20 µL of spores of *B. cereus* (OD_600_ = 1.0 ± 0.02) were lyophilized in microcentrifuge tubes. One nutrient disc was added to each tube of lyophilized spores. Then, 80 µL of the negative control and positive control were added into the tubes, which were vortexed for 15 s to mix the contents of each tube. After mixing, 15 µL of solution was filled into each micro-well on the well chip, which was then incubated for 30 min at 37 °C to induce expression of the marker enzyme in germinating spores. It should be noted that this standardization of incubation time is critical for ensuring that color intensity values are directly comparable between samples and between experimental runs.

Step 2—Enzyme–Substrate Reaction: After exposure, for the enzyme–substrate reaction, pillars on the pillar chip with substrate functionalized test papers attached were coupled with the wells, which were then incubated at 37 °C for 30 min. The pillar chip was inspected with the miniaturized microscopic imaging device for qualitative examination. Finally, the digital images were further analyzed to give a quantitative readout for each pillar area using the image analysis method described above. The presence of β-lactam antibiotics in milk was shown by the formation of a red color on the pillars, whilst the absence of β-lactam antibiotics was indicated by a yellow color when examined with both the naked eye qualitatively and the digital image analysis technique quantitatively. Importantly, the incubation times for both Step 1 and Step 2 were fixed at 30 min per step in all experiments, and all color readings were recorded at the end of this fixed incubation period to ensure directly comparable, standardized results across samples and experimental runs. The total assay time of approximately 60 min was selected as the optimal balance between sufficient color development for reliable detection and practical throughput requirements.

It is important to assess the greenness of the analytical method while developing the protocol, particularly in terms of waste generation, energy consumption, and the use of hazardous chemicals. While the CHARM-ROSA method, a known antibiotic detection test, remains a highly accurate and AOAC-approved gold standard, it operates as a single-point assay and therefore generates relatively higher plastic waste per sample.

In contrast, the proposed Biosensor-on-Pillar platform provides a greener alternative by enabling integrated, multi-sample analysis within a miniaturized and low-power device suitable for real-time, on-site applications. In terms of material usage, a typical CHARM-ROSA test employs a disposable plastic cassette weighing approximately 1.0–1.5 g per sample, whereas the Biosensor-on-Pillar utilizes a compact PMMA chip (~1.44 g) capable of processing multiple samples simultaneously. This translates to an effective plastic reduction of approximately 0.84–1.34 g per test, corresponding to a ~85–87% decrease in plastic usage on a per-sample basis.

At a larger scale, the environmental benefit becomes more pronounced. For instance, when analyzing 100 milk samples, the Biosensor-on-Pillar platform generates only ~16 g of plastic waste, compared to ~100 g produced by the conventional CHARM-ROSA method. These results clearly demonstrate the improved sustainability profile of the proposed system.

### 2.4. Determination of LODs for 12 β-Lactam Antibiotics

The LOD is the lowest concentration of β-lactam antibiotics that can induce detectable levels of the targeted enzyme expression in an enzyme induction-based test. The optimized assay was examined for LODs of 12 β-lactam antibiotics, namely, penicillin, amoxicillin, ampicillin, Oxacillin, Cloxacillin, Nafcillin, Carbenicillin, Cephalothin, Cefoxitin, Cefazolin, Cephalexin, and Cefuroxime. Milk samples were spiked with each antibiotic at concentrations below and above their maximum residue limits (MRLs) and then tested with the Biosensor-on-Pillar platform developed for the determination of LODs. The antibiotic detection results were also confirmed using AOAC-approved CHARM-ROSA strips. A great agreement was found between these two methods, validating the Biosensor-on-Pillar platform developed. The LOD of each β-lactam antibiotic in the developed test was defined as the lowest antibiotic concentration producing a visually detectable and quantitatively measurable color change, confirmed through ImageJ analysis as a mean red-channel intensity exceeding the negative control mean by at least three standard deviations (mean + 3σ criterion). Each concentration level was tested in at least duplicate to ensure reproducibility. The results of LODs of various β-lactam antibiotics are summarized in Table 1 and depicted in Supplementary Material Figure S5.

It should be noted that the LOD values presented in Table 1 were derived from a multi-stage experimental approach. (i) Initial screening: Multiple concentrations of 12 different β-lactam antibiotics were tested in spiked milk to identify the range at which color development occurs. (ii) Replication: Once the preliminary LOD was estimated, tests were performed in at least duplicate to ensure the consistency of both visual and quantitative (digital) red color development.

As can be seen from the results, the lowest LOD of 1 ppb was found with amoxicillin, whilst the majority were at or below their respective MRL. Two antibiotics (Cefalexin and Cefazoline) had significantly higher LODs than their MRLs, where the highest LOD was 1000 ppb for Cefalexin. These high levels of LODs were likely associated with the chemical structures of these compounds which may either hinder the enzyme–substrate reaction or affect the colorimetric process. The exact mechanism still remains to be fully understood.

Regarding cross-selectivity, the Biosensor-on-Pillar platform is designed as a group-specific screening tool rather than a compound-specific one. The fundamental working principle relies on the induction of the β-lactamase enzyme in germinating *Bacillus cereus* spores, which is specifically triggered by the presence of the β-lactam ring common to this class of antibiotics.

The cross-reactivity of the developed assay toward non-β-lactam antibiotics was not evaluated in the present study, as this aspect has been comprehensively addressed in our previous work [11]. In that study, the selected spore-based system demonstrated high specificity, exhibiting no observable interference in the presence of other classes of antibiotics. These findings confirm the robustness and selectivity of the assay for β-lactam antibiotics, even in complex matrices containing diverse antimicrobial agents.

### 2.5. Blind Sample Testing

**Figure 4:**
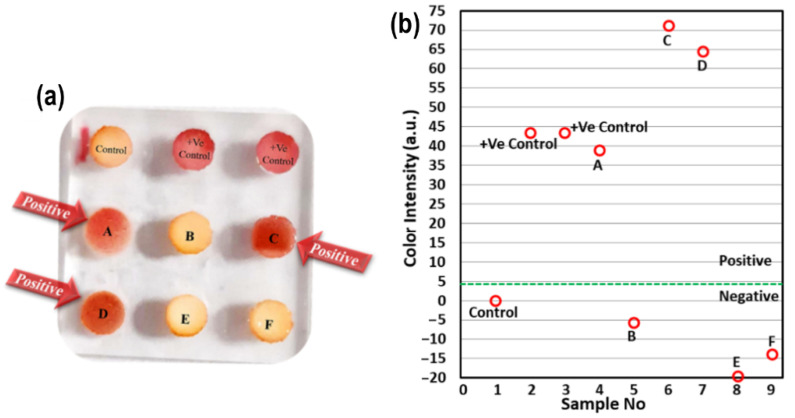
(**a**) Six blind sample tests with Biosensor-on-Pillar together with one negative control and two positive controls. (**b**) Color intensity readings of nine samples. (“−ve” indicates a negative result, where the milk is free from residues (or below the detection limit), and “+ve” indicates a positive result, where antibiotic residues are present in the milk sample at or above the detection limit (LOD)).

Using the optimized assay protocol, six blind milk samples were tested with the Biosensor-on-Pillar platform developed, together with penicillin G (4 ppb)-spiked milk samples (two positive controls) and a negative control (antibiotic-free raw milk). These samples were also simultaneously tested with the AOAC-approved CHARM-ROSA test for validation [11,17]. Figure 4 displays the readout of the nine samples on one single chip. It clearly shows red color development in samples containing β-lactam antibiotics, while the color of samples without antibiotics (or below LOD) remained comparable to the negative control.

Table 2 compares the test results from the Biosensor-on-Pillar detection with those from the existing CHARM-ROSA method. While the test results can be judged by eye, the ROSA Reader provides a more reliable and unbiased evaluation by measuring the optical density (OD) of the assay. It compares the intensity of the Test Line with that of the Control Line, reducing the chances of subjective interpretation. If this ratio drops below a set cut-off value—indicating a weaker Test Line—the sample is considered positive. Importantly, both the Biosensor-on-Pillar platform and the CHARM-ROSA method showed complete qualitative agreement (positive/negative) across all tested samples, including controls and blind milk samples. For clarity: the ‘Biosensor-on-Pillar Readings’ column reports the mean red-channel intensity change (8-bit ImageJ value, change in intensity vs. negative control); positive values indicate red color development consistent with antibiotic presence, while values near zero indicate no reaction. The ‘CHARM-ROSA Readings’ column reports the raw reader output from the ROSA device, where positive values indicate the presence of antibiotic residues (Test Line signal above cut-off) and negative values indicate a confirmed negative result (Test Line signal below cut-off). It was revealed that both approaches yielded agreeable results, indicating that the Biosensor-on-Pillar platform can be used for high throughput analysis in the field and dairy industries.

After a comparative analysis of some recent reports published on the detection of β-lactam antibiotics with the proposed assay, as shown in Table 3, it is possible to conclude that the method developed in this study has great potential for use in rapid, cost-effective, and high-throughput analysis of milk samples for the presence of β-lactam antibiotics in dairy business operators.

As can be seen, a comparison with the commercial gold standard, the CHARM-ROSA test, highlights several advantages of our Biosensor-on-Pillar platform. Despite an assay time of approximately 60 min, the nine-pillar configuration enables the simultaneous analysis of multiple samples, significantly improving throughput. The platform demonstrated strong agreement with the CHARM-ROSA method when validated using blind milk samples, confirming its reliability.

In addition, the use of a custom-built, 3D-printed miniaturized incubator reduces the overall cost and enhances portability, making it suitable for resource-limited dairy settings. Unlike conventional qualitative dipsticks, the system provides quantitative results through integrated imaging and ImageJ analysis. Furthermore, the scalable pillar-on-well design offers potential for increased throughput in large-scale dairy applications.

## 3. Material and Methods

### 3.1. Chemicals and Reagents

The propagation medium was prepared following the protocol outlined in our recent work [15]. The stock solution of the chromogenic substrate (nitrocefin) was prepared using the protocol described in our recent work [11] (Sigma Aldrich, Saint Louis, MO, USA; HiMedia, Mumbai, India). Standard solutions of β-lactam antibiotics (HiMedia) were prepared by serial dilution of the standard stock solution in milk and stored at −20 °C. Potassium phosphate buffer (HiMedia, India) (pH 6.8) was prepared following the protocol from our previous work [11] and stored at room temperature. Milk samples were procured from dairy farms near Karnal, India, and stored in a refrigerator without additional treatment before testing. Prior to use, milk samples were screened with the AOAC-approved CHARM-ROSA test to confirm their antibiotic-free status; only confirmed-negative samples were used as negative controls or as the base matrix for spiking experiments. The assay employs a standardized *B. cereus* indicator strain at a controlled density (OD_600_ = 1.0 ± 0.02), several orders of magnitude above any endogenous spore contamination in raw milk; consistent negative-control results confirmed the absence of background β-lactamase activity from endogenous microbiota.

### 3.2. General Instrumentation

A biosafety level-II cabinet (Esco Biotech Pvt. Ltd., Mumbai, Maharashtra, India) was used for the inoculation and reconstitution of spores. The sporulation medium was incubated in an incubator shaker (Eppendorf, Enfield, CT, USA). Centrifugation (Eppendorf, USA) was performed to separate the spore pellets, which were then stored in a deep freezer at −20 °C (Bluestar, Mumbai, Maharashtra, India). The spores were lyophilized using a lyophilizer (Labconco, Kansas City, MO, USA), and their optical densities (ODs) were adjusted using a microbiological plate reader (PerkinElmer, Shelton, CT, USA). Finally, the chromogenic substrate was immobilized on the paper using an Eppendorf micro-pipette (USA).

### 3.3. Preparation of B. cereus Spores

The procedure used in our previous study [11] for *B. cereus* spore production was followed. A single pure colony was transferred to a propagation medium and incubated at 37 °C for 24 h. The culture was then inoculated into a growth medium tryptone glucose yeast extract (TGY) broth (0.5% tryptone, 0.25% yeast extract, 0.1% dextrose at pH 7) at 1% and incubated at 37 °C for 48 h. Subsequently, the culture was inoculated into a sporulation medium at 7.5% to induce spore production. Incubation was carried out at 37 °C for 42 h, followed by spore harvesting through centrifugation at 10,000 rpm for 10 min at 10 °C. The spore pellet was washed twice with potassium phosphate buffer (pH 6.8). The cleaned pellet was then resuspended in a known volume of the same buffer, and the suspension was standardized to an optical density (OD_600_) of 1.0 ± 0.02 using a microbiological plate reader. The obtained spore suspension was subsequently analyzed for total viable count and spore count. For spore lyophilization, 20 μL of the final spore solution was loaded into microcentrifuge tubes and lyophilized at −84 °C under a vacuum of 1 ± 0.5 torr (1 Torr = 133.33 Pascal) for 1 h. The lyophilized spores were then packed in a plastic bag and stored at 4 °C [15].

### 3.4. Design and Fabrication of the Biosensor-on-Pillar Platform

The Biosensor-on-Pillar platform consisted of three main subsystems: (a) a pillar/well chip, (b) miniaturized incubator, and (c) miniaturized microscopic imaging.

**Figure 5:**
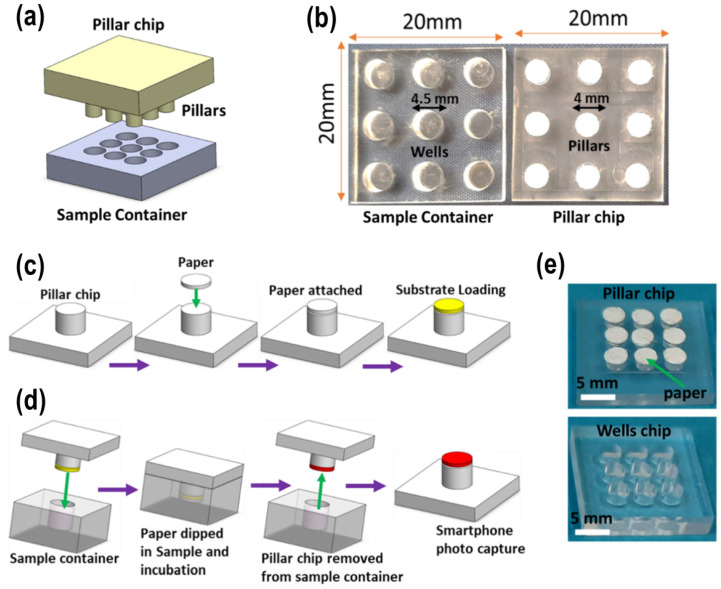
(**a**) Schematic design of the Biosensor-on-Pillar chips. (**b**) Photograph of the pillar/well chips. (**c**) Design and fabrication procedure of pillar chips. (**d**) Working principle of the Biosensor-on-Pillar chips. (**e**) Photograph of Biosensor-on-Pillar chips.

(a) Pillar/well chips: The chips were designed using AutoCAD 2018 software, with nine pillars of 4 mm diameter × 1 mm height on the pillar chip and nine wells of 4.5 mm diameter × 2.4 mm depth on the well chip (Figure 5). The dimensions of the chip were 20 × 20 × 3 mm (width × length × height). Both chips were fabricated from a poly(methyl methacrylate) (PMMA) sheet using an LPKF ProtoMat S100 micro-milling machine (Osteriede 7, Garbsen, Germany) [31,32]. Due to its higher speed and flexibility, the milling process was selected over traditional chemical etching methods. Additionally, the fabrication time for each pillar chip was under 30 min, and the parameters could be easily modified using the system application on the computer. The surface of the chip was first cleaned with compressed air to remove dirt and debris, then rinsed with deionized water and ethanol, respectively. The chips were cleaned again using compressed air to dry them. Filter papers (Whatman Grade 3, 4 mm diameter) were attached to the pillars with double-sided tape. The plates were then sealed in clean Petri dishes for storage to prevent contamination. The step-by-step Biosensor-on-Pillar fabrication procedure is shown in Figure 5c.

(b) Miniaturized incubator: The miniaturized incubator was designed using SolidWorks 2018 (as shown in Supplementary Material Figure S1) and printed in black poly(lactic acid) (PLA) material using an Ultimaker-2 3D printer [33]. The incubator consisted of an incubation chamber, lid, and temperature controller (Figure S1). The heating plate (RS PRO Heater Mat, 2.5 W, 50 × 50 mm, 12 V) was fixed inside the incubation chamber to provide continuous heat for the incubation of samples inside the well chips and reagents on the pillars. The heating plate can provide temperatures up to 100 °C. It was connected to the Carel IR33 Panel Mount PID Temperature Controller (Brugine PD, Italy), which has dimensions of 76.2 × 34.2 mm and provides two output relays, requiring a 24 V DC supply voltage. In our experiments, the temperature was set to 37 °C for all steps. A thermocouple (Carel Type NTC, 10K) was fixed on the plate to accurately measure and maintain the temperature. The thermocouple had a temperature range of −50 to 50 °C. The working principle and image of the miniaturized incubator are illustrated in Figure S1.

(c) Miniaturized microscopic imaging: The miniaturized microscopic imaging system was designed using SolidWorks (Figure S2) and printed in black poly(lactic acid) (PLA) material using an Ultimaker-2 3D printer. The detector consisted of a mini microscope (RS PRO USB Digital Microscope, 2M pixels, 20 → 200× magnification) (Corby, Northants, UK) attached to the black box and a pillar chip holder for sliding the chips into the detection area (Figure S2). The pillar chip was placed on the holder and slid into the box, where the camera captured images. Images of the pillar chips were captured using the miniaturized USB digital microscope and subsequently analyzed using ImageJ (version ij154-win-java8, NIH, 9000 Rockville Pike Bethesda, MD, USA). Quantification was performed by extracting the mean red-channel intensity from 8-bit images of each pillar area (pixel values on a 0–255 scale). Each pillar’s region of interest (ROI) was defined as the circular area of the paper disc, and the reported “reading” represents the mean pixel intensity change relative to the negative control [32,34].

### 3.5. Optimization of Assay Conditions for Detecting β-Lactam Antibiotics in Milk

Key assay parameters, including sample volume, substrate volume, and incubation time and temperature, were optimized using penicillin antibiotics as a positive control to identify the optimal combination of these parameters for detecting the β-lactam group in milk using the Biosensor-on-Pillar analysis.

Milk samples were spiked with amoxicillin antibiotic and incubated with spores and nutrients to induce β-lactamase production in germinating spores, as previously demonstrated [11,18]. Briefly, milk samples were incubated with spores and nutrients at 37 °C in sample wells (30 min), and the pillar chip was dipped into the samples, followed by 30 min incubation with substrate before the final imaging (Figure S4a). The detection of antibiotic concentration was also tested by paper strips developed at NDRI, India and a great agreement was found between these two methods (Figure S4b).

The concentration of *B. cereus* spores was standardized to an optical density (OD_600_) of 1.0 ± 0.02. Prior to the assay, the spores were combined with one nutrient disc (paper disc containing tryptone) per tube and reconstituted in 80 µL of milk [11], with a total volume ranging from 7 to 17 μL, filled into the well. The amount of chromogenic substrate nitrocefin loaded onto the test paper varied between 1.25 μg and 3.75 μg. The incubation temperature was controlled, ranging from 32 to 37 °C for 30 min. A stepwise protocol for the detection of the β-lactam group in milk is illustrated in Supplementary Material Figure S3. To verify the enzyme induction concept, an assay was performed using the Biosensor-on-Pillar for the detection of β-lactam antibiotics in milk. Firstly, spores of *B. cereus* were placed in two microcentrifuge tubes, and nutrients were added to each tube. A negative control (antibiotic-free milk) and a positive control (penicillin-containing milk) were added to the respective tubes, and vortexed for 15 s to mix the contents. After mixing, the samples were placed into the micro-wells on the chip and incubated for 30 min at 37 °C to induce the expression of the marker enzyme in germinating spores. Subsequently, the pillar chip with substrate-functionalized paper was coupled with the well chip, which was then incubated at 37 °C for 30 min. This resulted in red color development on the test paper, indicating the induction of the β-lactamase enzyme in germinating spores, while the yellow color remaining in the control sample indicated the absence of the β-lactamase enzyme.

Initially, the β-lactam antibiotic standards were prepared at concentrations both below and above their MRLs (FSSAI/CODEX) in milk [35] following the optimized assay protocol. The minimum concentration at which a detectable color change (red) on the pillar paper was recorded was considered the LOD (limit of detection) of the Biosensor-on-Pillar analysis. Parallel tests were also conducted using the CHARM-ROSA method [11,17,18] to validate the results obtained from the Biosensor-on-Pillar analysis.

## 4. Conclusions

A spore-based Biosensor-on-Pillar miniaturized platform was developed for detecting β-lactam antibiotics in milk, employing an enzyme induction-based principle. The platform consisted of three main subsystems, namely, a pillar/well chip, miniaturized incubator, and miniaturized microscopic imaging. Key operating parameters and assay conditions were optimized to establish an efficient assay protocol, including the sample volume, substrate volume, incubation time and temperature. The platform was then characterized by determining the limits of detection (LODs) for 12 β-lactam antibiotics in accordance with regulatory standards. The developed biosensor demonstrated a sensitivity of ≤MRL for β-lactam antibiotics, as recommended by FSSAI/CODEX [8]. Furthermore, six blind milk samples were tested alongside β-lactam antibiotic-spiked milk samples (two positive controls) and a negative control. The results were validated against the CHARM-ROSA method, showing high agreement within one hour. The platform also exhibited multiplex capability, allowing for the high-throughput analysis of nine samples in a single run, with the potential for further expansion by fabricating additional pillars and wells on a single chip. Additionally, the assay time could potentially be reduced by integrating the device with electrochemical tools. The developed technology offers a low-cost, sensitive, selective, and reproducible approach for real-time detection of β-lactam antibiotics in milk, making it particularly suitable for resource-limited settings.

## Figures and Tables

**Table 1:** Regulatory standards for different antibiotics and their limit of detection (LOD) results.

Antibiotic	MRL	LOD
Amoxicillin	4 ppb (FSSAI)	1 ppb
Ampicillin	10 ppb (FSSAI)	2 ppb
Penicillin	4 ppb (FSSAI)	2 ppb
Carbenicillin	Not specified	10 ppb
Cloxacillin	30 ppb (EU)	10 ppb
Nafcillin	30 ppb (EU)	10 ppb
Oxacillin	30 ppb (EU)	20 ppb
Cefalexin	10 ppb (FSSAI)/100 ppb (EU)	1000 ppb
Cephalothin	Not specified	10 ppb
Cefoxitin	Not specified	10 ppb
Cefuroxime	Withdrawn (prev. 50 ppb, EU); no current MRL	100 ppb
Cefazoline	50 ppb (EU)	300 ppb

**Table 2:** Comparative results of samples using all three methods used in the study.

Sample	Biosensor-on-Pillar Readings	Biosensor-on-Pillar Results	CHARM-ROSA Readings
−ve Control	0	−ve	−662
+ve Control	+43.458	+ve	+3635
+ve control	+43.478	+ve	+3592
A	+38.859	+ve	+1365
B	−5.579	−ve	−600
C	+71.188	+ve	+2372
D	+64.491	+ve	+2125
E	−19.586	−ve	−717
F	−13.921	−ve	−590

Note: “−ve” indicates a negative result, where the milk is free from residues (or below the detection limit), and “+ve” indicates a positive result, where antibiotic residues are present in the milk sample at or above the detection limit (LOD).

**Table 3:** Comparison of some recent reports published on the detection of β-lactam antibiotics with the proposed assay.

Sample No.	Sensor Type	Sensing Element	Target Antibiotic	LODs	Ref.
1	Electrochemical	Graphite-based conductive ink in paper-based electrodes	Amoxicillin	3654 ppb	[24]
2	Immuno-chromatographic assay	Monoclonal antibodies	β-lactam antibiotics	1–100 ppb	[25]
3	Electromembrane extraction	Capillary zone electrophoresis	Benzylpenicillin, amoxicillin, and ampicillin	3–100 ppb	[26]
4	Single-drop electrochemical immune sensor	Amoxicillin antibodies conjugated magnetic nanoparticles	Amoxicillin	161 ppb	[27]
5	Electrochemical receptor sensor	Graphene/thionine (GO/TH) composite and horseradish peroxidase-labelled ampicillin	Cefquinome, cefalexin, cefquinoxime, cefotafur, penicillin G and ampicillin	0.13–41.45 ppb	[28]
6	Microfluidic paper-based analytical device	Metallochromic complexes	β-lactam antibiotics	5000 ppb	[29]
7	Molecularly imprinted polymer-based optical sensor	Surface Plasmon Resonance (SPR)	Penicillin G	5 ppb	[30]
8	Spore-based paper-strip biosensor	Bacterial spores	β-lactam antibiotics	5–1000 ppb	[11]
9	Present study	Bacterial spores	β-lactam antibiotics	5–1000 ppb	

## Data Availability

All data generated or analyzed during this study are included in this published article and its Supplementary Materials.

## Supplementary Materials

The following supporting information can be downloaded at: https://www.mdpi.com/article/10.3390/molecules31091436/s1. Figure S1: Miniaturized incubator; Figure S2: Miniaturized microscopic imaging; Figure S3: Stepwise protocol for the detection of β-lactam group antibiotics in milk; Figure S4: (a) Photo showing incubation of nitrocefin. (b) Representative examples of the same samples incubated in paper strips; Figure S5: Detection limits obtained for β-lactam group of antibiotics.
