# Supplementary material for: A Spore-Based Biosensor-on-Pillar Platform for Detecting β-Lactam Antibiotics in Milk
Source: Molecules. 2026 Apr 26;31(9):1436. doi: 10.3390/molecules31091436 (PMC13164989; doi:10.3390/molecules31091436)
Supplement: Supplementary file 1 [file molecules-31-01436-s001.zip › molecules-4188758-supplementary.pdf]

<u>**Supporting Information**</u>

A Spore-Based Biosensor-on-Pillar Platform for Detection of ß-Lactam Antibiotics in Milk

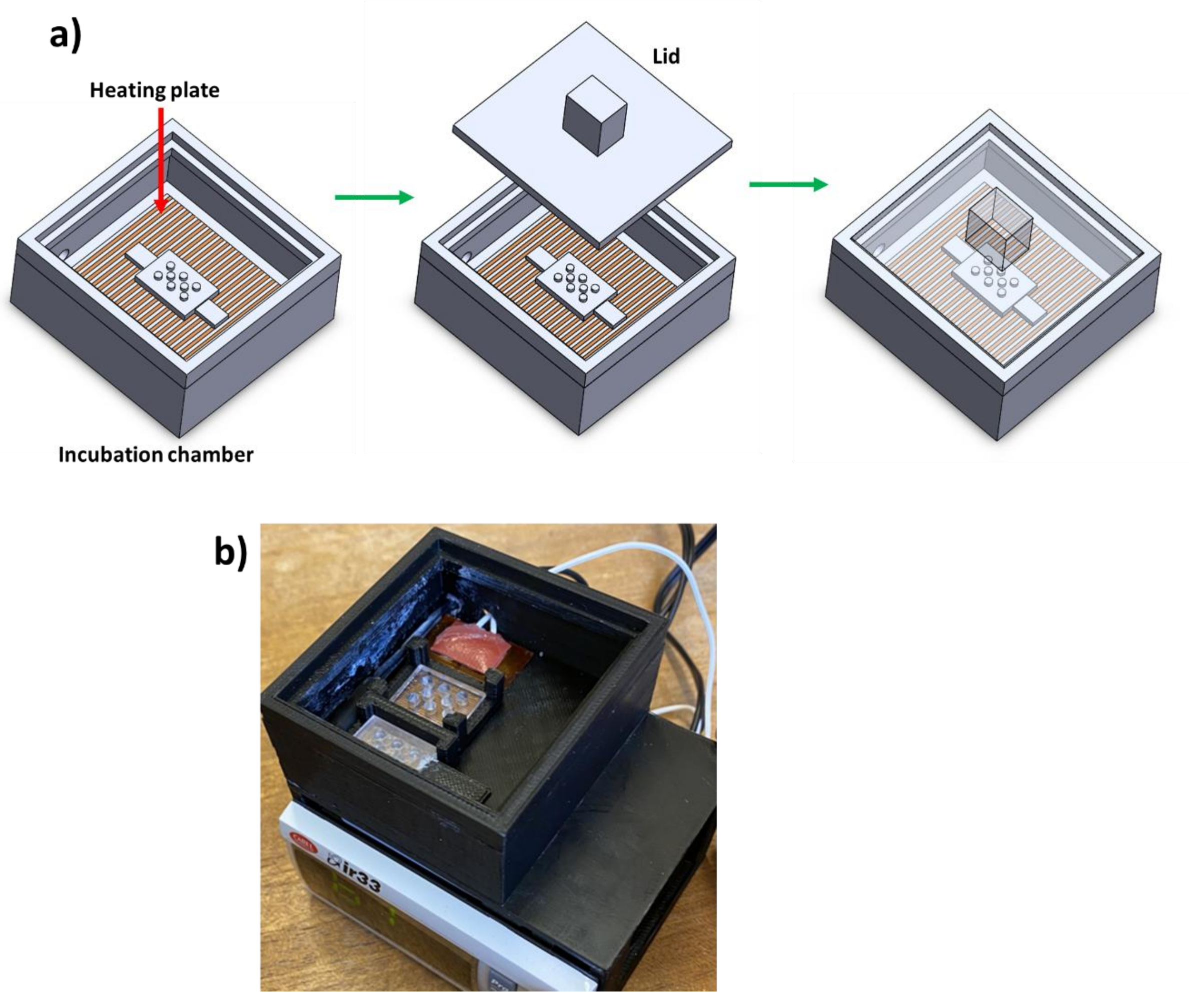

Figure S1. Miniaturized incubator: The miniaturized incubator was designed using SolidWorks and printed in black poly(lactic acid) (PLA) material using an Ultimaker-2 3D printer. The incubator comprised an incubation chamber, lid, and temperature controller (a). The heating plate (RS PRO Heater Mat, 2.5 W, 50 x 50 mm, 12 V) was fixed in the incubation chamber to provide continuous heat for the incubation of samples inside well chips and reagents onto the pillars. The heating plate can provide temperatures up to 100 °C. The plate was connected with the Carel IR33 Panel Mount PID Temperature Controller, with dimensions of 76.2 x 34.2 mm, providing two output relays and required 24 V dc supply voltage. The temperature sensor, a thermocouple (Carel Type NTC, 10K), was also fixed on the plate to accurately measure and maintain the temperature on the plate, with a range of -50 to 50 °C. The photograph of the miniaturized incubator (b).

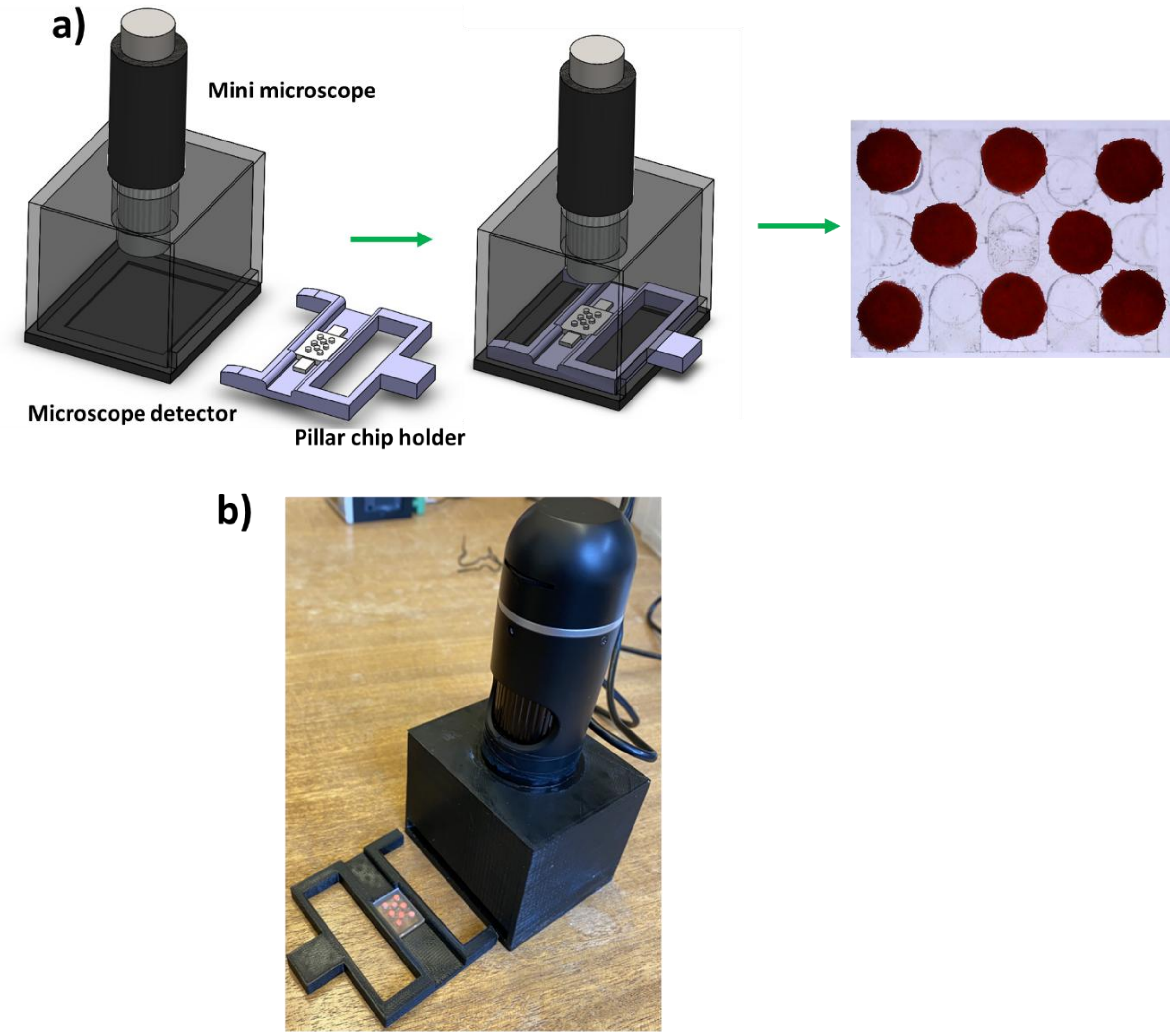

Figure S2. Miniaturized microscopic imaging: The miniaturized microscopic imaging platform was designed using SolidWorks (a) and printed in black poly(lactic acid) (PLA) material using an Ultimaker-2 3D printer (b). The detector consisted of a mini microscope (RS PRO USB Digital Microscope, 2M pixels, 20 → 200X Magnification) attached to the black box and a pillar chip holder for sliding the chips into the detection area. The pillar chips were placed on the holder and slid into the box where the camera took images. The colour image was read by image analysis software (ImageJ, NIH), and the colour intensity was measured for quantification.

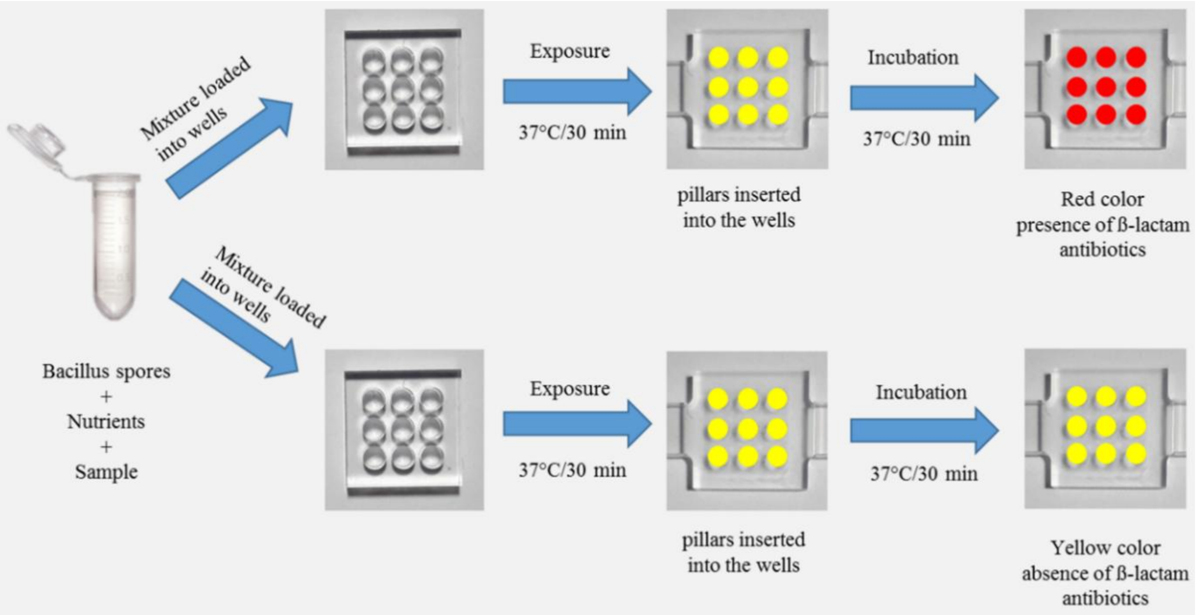

Figure S3. Stepwise protocol for the detection of β-lactam group antibiotics in milk.

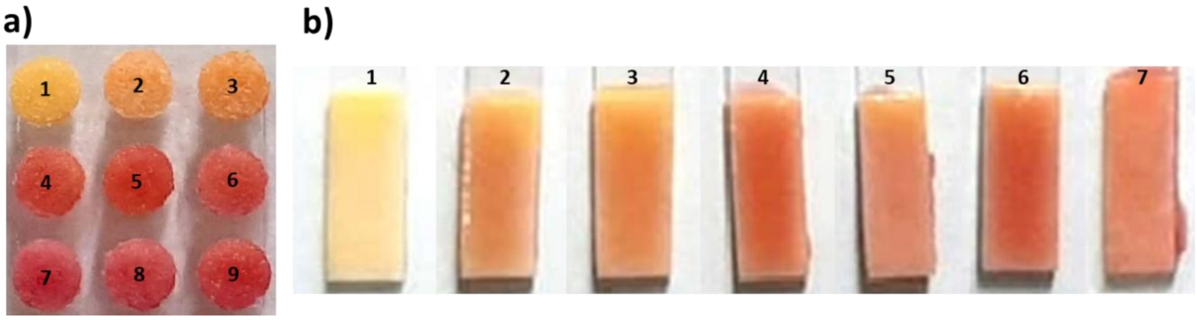

Figure S4. (a) Photo showing incubation of nitrocefin (1 mg/mL) coated paper with different concentrations of amoxicillin (Pillar **1-** 0 ppb; **2-** control milk; **3-** 1 ppb; **4-** 2 ppb; **5-** 3 ppb; **6-** 4 ppb; **7-** 5ppb; **8-** and **9-** 10 ppb). (b) Representative examples of the same samples incubated in paper strips (Strip **1-** control milk; **2-** 1 ppb; **3-** 2 ppb; **4-** 3 ppb; **5-** 4 ppb; **6-** 5 ppb; **7-** 10 ppb).

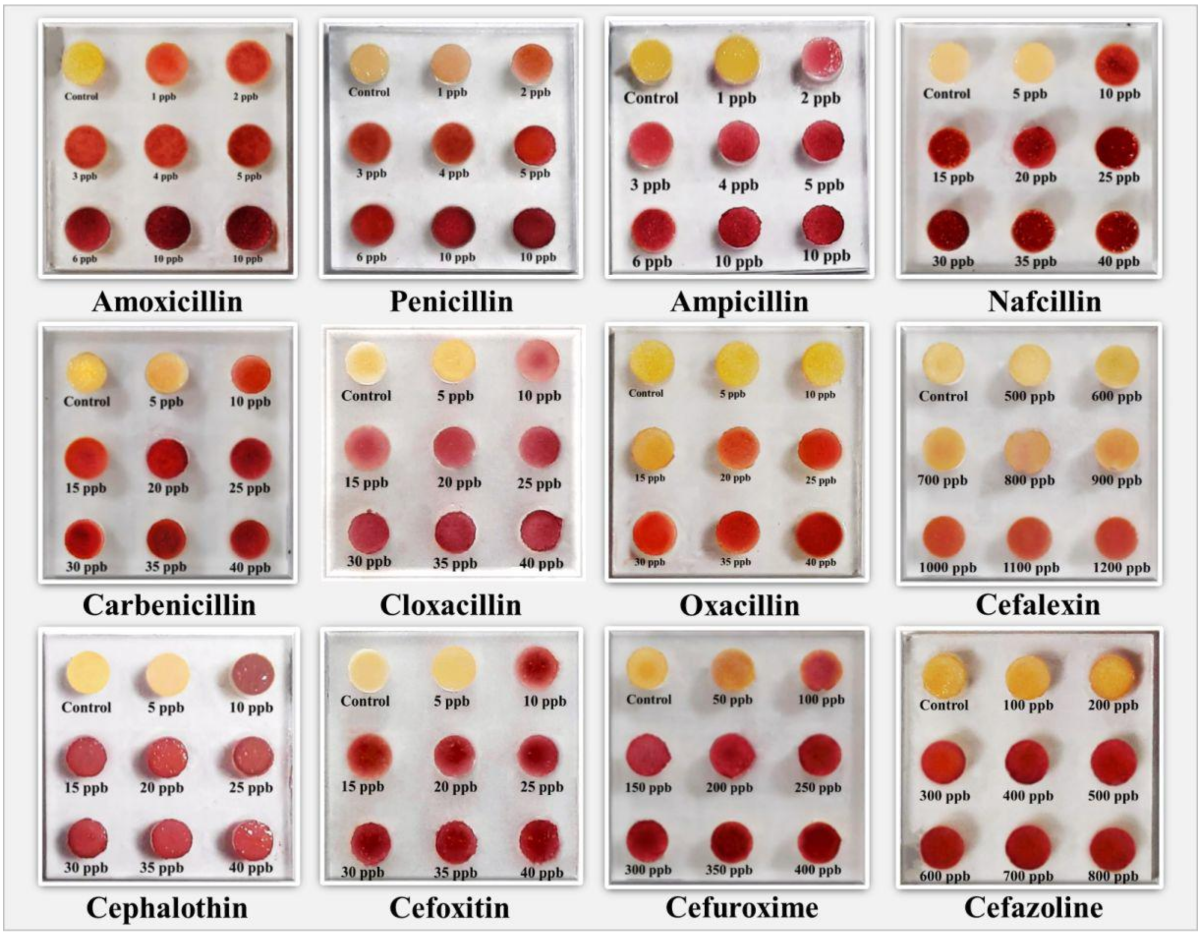

Figure S5. Detection limits obtained for ß-lactam group of antibiotics.
